# Therapeutic Potential of Baicalein in Endometrial Cancer: Suppression of mTOR Signaling and Synergy with Metformin

**DOI:** 10.3390/ijms262211061

**Published:** 2025-11-15

**Authors:** Alison L. Dumitriu, Wei Wen, Jin Yan, Quanhua Xing, Finn E. Ro, Ernest S. Han, John H. Yim

**Affiliations:** 1 Division of Surgical Oncology, Department of Surgery, City of Hope National Medical Center, Duarte, CA 91010, USA; alisondumitriu@llu.edu (A.L.D.); wwen@coh.org (W.W.); jyan@coh.org (J.Y.); qxing@coh.org (Q.X.); finneverro@gmail.com (F.E.R.); 2 Division of Gynecologic Oncology, Department of Surgery, City of Hope National Medical Center, Duarte, CA 91010, USA; ehan@coh.org

**Keywords:** endometrial cancer, baicalein, metformin, AMPK/PI3K/mTOR pathway, combination, synergy

## Abstract

Baicalein, a natural flavonoid derived from traditional medicinal herbs, has demonstrated anticancer activity in various malignancies, but its role in endometrial cancer remains largely unexplored. In this study, we investigated the therapeutic potential of baicalein, alone and in combination with metformin, in human endometrial cancer cells. Given that the mTOR signaling pathway is frequently dysregulated in endometrial cancer due to PTEN loss, we examined how baicalein affects this pathway. Our results demonstrated that baicalein significantly inhibited cell proliferation in a dose-dependent manner, which was associated with increased DDIT4 expression, activation of AMPK, and decreased phosphorylation of mTOR downstream targets S6K1 and S6. In vivo, baicalein treatment led to a reduction in tumor volume in HEC-1A xenograft female nude mice without affecting body weight. While metformin also reduced cell viability, baicalein achieved comparable effects at lower concentrations. The combination of baicalein and metformin produced a synergistic anti-tumor effect and more effectively inhibited the AMPK/PI3K/mTOR signaling pathway than either agent alone. These findings suggest that baicalein may represent a promising, non-toxic therapeutic option for endometrial cancer, particularly when used in combination with metformin. Further investigation is warranted to assess the clinical relevance of this strategy.

## 1. Introduction

Endometrial cancer is the most common gynecologic malignancy in the United States, with both its incidence and mortality rates steadily increasing [1,2,3,4]. Alarmingly, death rates have more than doubled over the past two decades [5], underscoring the ur-gent need for more-effective treatment options. Many patients with endometrial cancer are older and frequently have comorbid conditions such as obesity, diabetes mellitus, and hypertension, making the development of low-toxicity therapies particularly challenging [2,3]. In this context, non-toxic natural supplements represent a potential alternative. Among these, dietary flavonoids have emerged as promising candidates, recognized for their potent anti-cancer activities [6,7,8].

Baicalein, a naturally occurring flavonoid found in thyme and in traditional Asian medicinal herbs such as *Scutellaria baicalensis* (Huang Qin), has demonstrated a favorable safety profile and appears to be non-toxic in both humans and animals [9,10,11]. It has also been extensively studied for its therapeutic potential in a variety of conditions, including cancer, metabolic disorders, and inflammatory diseases [9,10,12]. While its anti-cancer properties have been characterized across a range of malignancies, including liver, breast, lung, cervical, thyroid, gastric, bladder, and pancreatic cancers, its potential effects in endometrial cancer remain largely unexplored [11,13,14]. Given its favorable safety profile and promising anticancer properties, baicalein warrants further investigation as a potential treatment for endometrial cancer.

Previous studies have demonstrated that baicalein inhibits cancer cell proliferation by upregulating the expression of DNA-Damage-Inducible Transcript 4 (DDIT4), which in turn suppresses the phosphoinositide 3-kinase/protein kinase B/mammalian target of rapamycin (PI3K/AKT/mTOR) signaling pathway [11,15]. The PI3K/AKT/mTOR pathway is a central regulator of cell growth, survival, and metabolism and is frequently hyperactivated in numerous cancers. Dysregulation of this pathway often results from mutations in key components such as PI3K, AKT, or mTOR, or from the loss of tumor suppressors like phosphatase and tensin homolog (PTEN), leading to uncontrolled cell proliferation and survival [16]. As a result, the PI3K/AKT/mTOR pathway has become a key target in cancer therapy, driving considerable efforts to develop inhibitors that can disrupt its individual components to suppress tumor progression [17,18,19]. In endometrial cancer, alterations in the PI3K/AKT/mTOR pathway, often driven by PTEN loss, have established it as a critical therapeutic target. Numerous clinical trials are currently investigating the effectiveness of inhibitors targeting this pathway in endometrial cancer patients [20,21,22,23].

Recent research has increasingly highlighted the potential of metformin as an anti-cancer agent, particularly in endometrial cancer. Although traditionally used as a first-line treatment for hyperglycemia in patients with type 2 diabetes mellitus, metformin has demonstrated promising anti-tumor activity in preclinical studies across a variety of malignancies, including lung, breast, liver, and ovarian cancer and melanoma [24,25,26,27,28]. In the context of endometrial cancer, metformin has been shown to suppress cell proliferation, at least in part, by modulating key signaling pathways such as AMP-activated protein kinase (AMPK) and the PI3K/mTOR axis. Based on these findings, metformin is currently being evaluated in multiple phase II and III clinical trials to assess its efficacy and safety in patients with endometrial cancer [29,30,31,32].

In this study, we investigated the anti-cancer activity of baicalein, both alone and in combination with metformin, in endometrial cancer cells.

## 2. Results

### 2.1. Effect of Baicalein on Cell Growth in Endometrial Cancer Cells

**Figure 1:**
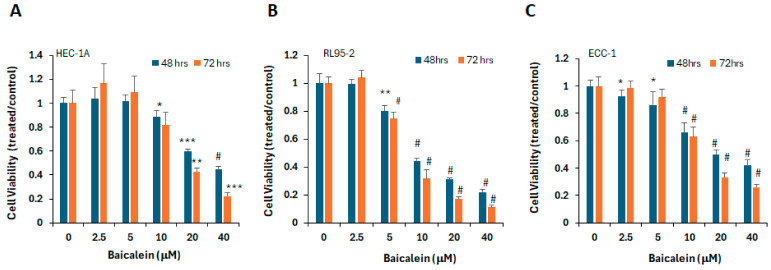
Baicalein reduces viability of endometrial cancer cells. (**A**) HEC-1A, (**B**) RL95-2, and (**C**) ECC-1 cells were treated with vehicle control (DMSO) or increasing concentrations of baicalein (2.5–40 μM) for 48 or 72 h. Cell viability was measured using the MTS assay. *, *p* < 0.05; **, *p* < 0.005, ***, *p* < 0.0005, #, *p* < 0.0001 for baicalein vs. vehicle.

To evaluate the anti-tumor activity of baicalein in endometrial cancer, we examined its effects on cell viability across several endometrial cancer cell lines, including HEC-1A, RL95-2, and ECC-1. Exponentially growing cells were treated with baicalein at concentrations ranging from 2.5 to 40 μM for 48 and 72 h. As shown in Figure 1, baicalein significantly reduced cell viability in a dose-dependent manner. These findings indicate that baicalein effectively suppresses the proliferation of endometrial cancer cells.

### 2.2. Effect of Baicalein on AMPK/PI3K/mTOR Signaling Pathway

**Figure 2:**
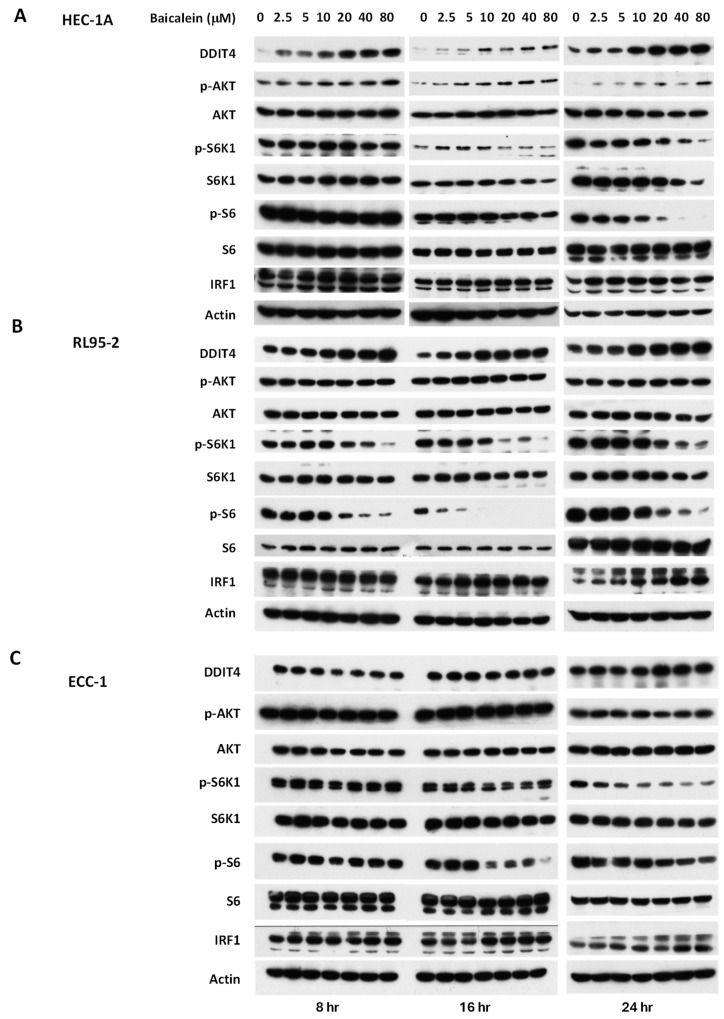
Impact of baicalein on the mTOR signaling pathway in (**A**) HEC-1A, (**B**) RL95-2, and (**C**) ECC-1 cells. Cells were treated with varying concentrations of baicalein for 8, 16, and 24 h. Whole-cell lysates were harvested, and expression levels of DDIT4, IRF-1, phosphorylated AKT kinase (p-AKT), phosphorylated S6 kinase (p-S6K1), and phosphorylated S6 (p-S6) were analyzed by Western blot.

To investigate the mechanisms underlying the anti-tumor activity of baicalein, we examined molecular changes in endometrial cancer cells following treatment. Previous studies have shown that baicalein induces the expression of DDIT4 and IRF-1 (Interferon regulatory factor-1), leading to inhibition of mTOR signaling in other cancer types [11,15]. To determine whether similar effects occur in endometrial cancer, we treated HEC-1A, RL95-2, and ECC-1 cells with baicalein at concentrations ranging from 2.5 to 80 μM for various durations and analyzed the expression of DDIT4 and IRF-1. DDIT4 expression was induced in a dose-dependent manner in HEC-1A and RL95-2 cells, but not in ECC-1 cells. Conversely, IRF-1 expression was significantly upregulated at 24 h in all three cell lines (Figure 2). Additionally, baicalein treatment resulted in reduced phosphorylation of mTOR downstream effectors (pS6K1 and pS6) across all three cell lines.

**Figure 3:**
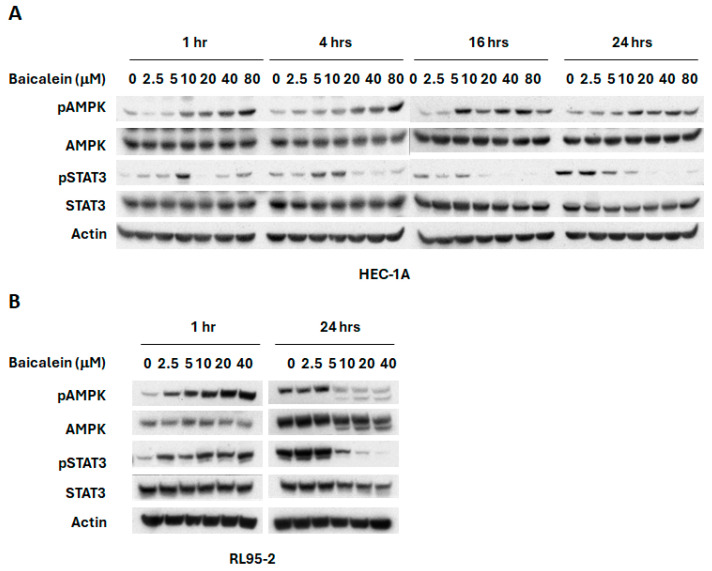
Effects of baicalein on AMPK and STAT3 signaling pathways. (**A**) HEC-1A and (**B**) RL95-2 cells were treated with varying concentrations of baicalein for different time points. Whole cell lysates were collected, and phosphorylation levels of AMPK (p-AMPK) and STAT3 (p-STAT3) were assessed by Western blot.

Activation of AMPK has been associated with suppression of the PI3K/mTOR signaling pathway [33,34,35,36]. To investigate whether baicalein modulates this axis, we examined its effect on AMPK activation. As shown in Figure 3, baicalein significantly increased AMPK phosphorylation in HEC-1A cells, suggesting that AMPK activation may contribute to its inhibitory effect on mTOR signaling. In addition, we assessed the Janus kinases/signal transducer and activator of transcription 3 (JAK/STAT3) pathway, which is constitutively activated in endometrial cancer and plays a key role in tumor growth and progression [37,38,39]. Our results demonstrate that baicalein significantly reduced phosphorylated STAT3 (pSTAT3) levels in HEC-1A cells in a dose and time dependent manner (Figure 3A). Similar activation of AMPK and inhibition of pSTAT3 were also observed in RL95-2 cells (Figure 3B), further supporting baicalein’s regulatory impact on these critical signaling pathways.

### 2.3. Anti-Tumor Activity of Baicalein in Endometrial Cancer Mouse Model

**Figure 4:**
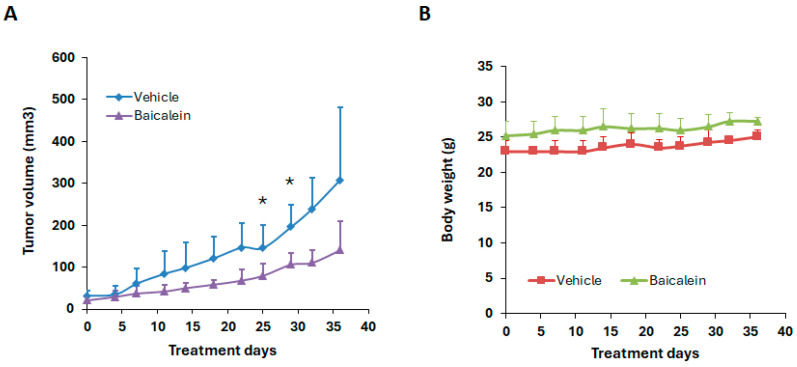
Effect of baicalein on tumor growth in the HEC-1A xenograft mouse model. Nude mice bearing subcutaneous HEC-1A tumors were treated with either vehicle control or baicalein at 80 mg/kg by oral gavage daily. (**A**) Tumor volume and (**B**) body weight were measured twice a week over the course of the treatment period. * *p* < 0.05, baicalein vs. vehicle.

We next evaluated the anti-tumor efficacy of baicalein in an HEC-1A xenograft mouse model (Figure 4). HEC-1A cells were implanted subcutaneously into the right flank of nude mice. Once tumors became palpable, mice were randomized into two groups to receive either vehicle control or baicalein, administered by intraperitoneal (i.p.) injection. Tumor volume and body weight were measured twice weekly throughout the treatment period. As shown in Figure 4, baicalein treatment resulted in a reduction of tumor volume compared to controls, while maintaining stable body weight, indicating minimal toxicity. Although a clear trend toward tumor suppression was observed, the difference did not reach statistical significance at all time points.

### 2.4. Combination of Baicalein with Metformin in Endometrial Cancer Cells

**Figure 5:**
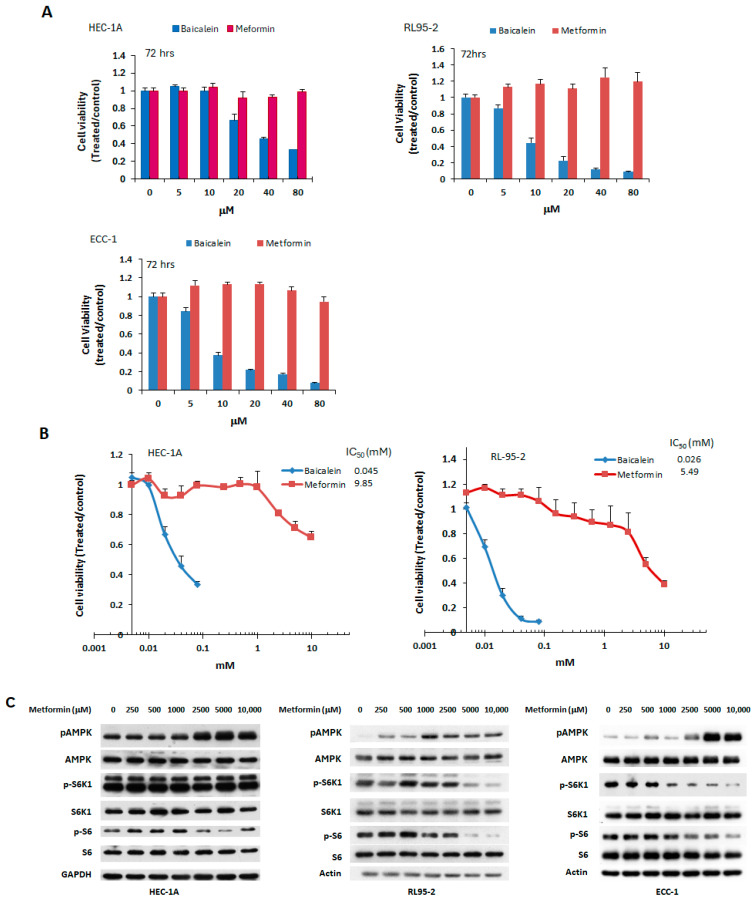
Effect of metformin on endometrial cancer cell viability and signaling pathways. (**A**) HEC-1A, RL95-2, and ECC-1 cells were treated with vehicle (DMSO), baicalein, or metformin at concentrations ranging from 2.5 to 80 μM for 72 h. (**B**) HEC-1A and RL95-2 cells were treated with metformin at concentrations between 2.5 μM and 10 mM for 72 h. Cell viability was assessed by MTS assay, and IC50 values were calculated using the Chou-Talalay method. Results are presented as a ratio relative to vehicle-treated controls (DMSO). (**C**) Cells were treated with varying concentrations of metformin for 24 h. Whole-cell lysates were collected, and phosphorylation levels of AMPK (p-AMPK), S6K1 (p-S6K1), and S6 (p-S6) were evaluated by Western blot.

Metformin, a well-known mTOR inhibitor through AMPK activation, has been previously studied for its potential in treating endometrial cancer [28]. In this study, baicalein was compared to metformin for its anti-tumor effects in endometrial cancer cell lines. HEC-1A, RL95-2, and ECC-1 cells were treated with increasing concentrations (5–80 μM) of either baicalein or metformin for 72 h. As shown in Figure 5, baicalein significantly reduced cell viability across all three cell lines, whereas metformin at the same concentrations had no appreciable effect on cell growth. Notably, metformin required over 200-fold higher (millimolar) concentrations to achieve growth inhibition. The IC_50_ values for baicalein and metformin were 0.045 mM vs. 9.85 mM in HEC-1A cells, and 0.026 mM vs. 5.49 mM in RL95-2 cells, respectively, indicating that baicalein is substantially more potent than metformin in suppressing endometrial cancer. Consistent with the cell viability results, a much higher concentration of metformin was needed to activate AMPK and inhibit mTOR downstream effectors (S6K1 and S6) in these cells (Figure 5C).

**Figure 6:**
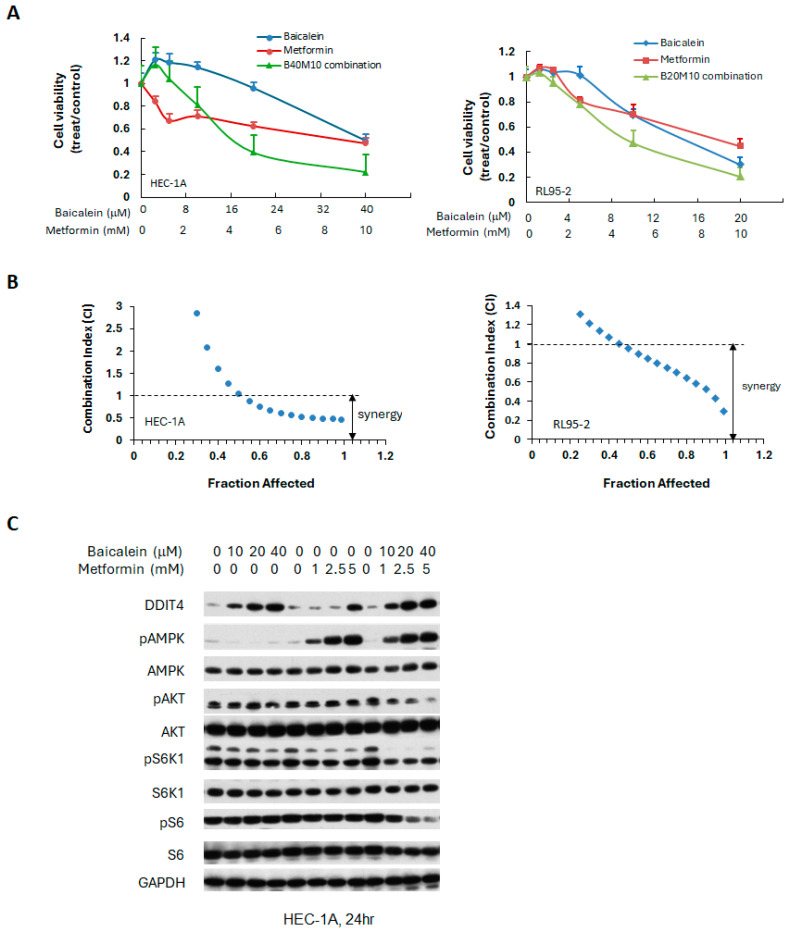
Synergistic effects of baicalein combined with metformin in human endometrial cancer cells. (**A**) Cells were treated with baicalein and metformin, alone or in combination at fixed molar ratios across various concentrations. Cell viability was assessed after 72 h. (**B**) The combination index (CI) was calculated at fraction affected (a fraction of cell viability inhibited) using the Chou-Talalay method to evaluate synergy. (**C**) HEC-1A cells were treated with indicated concentrations of baicalein and metformin, individually or combined, for 24 h. Western blot analysis was conducted to assess the levels of phosphorylated and total AMPK, S6K1, S6, and AKT. GAPDH served as the loading control.

Although both baicalein and metformin can activate AMPK phosphorylation, the mechanisms underlying AMPK activation may differ between these two as reported in other cells [40,41]. In addition to their effects on the AMPK/PI3K/mTOR signaling pathway, both agents have been reported to inhibit cancer cell proliferation by modulating other signaling cascades, including the JAK/STAT3, mitogen-activated protein kinase/ extracellular signal-regulated kinase (MAPK/ERK), and nuclear factor kappa-light-chain-enhancer of activated B cells (NF-κB) pathways. Therefore, the combination of baicalein and metformin may exert synergistic or enhanced anti-cancer effects. To investigate this possibility, HEC-1A and RL95-2 endometrial cancer cells were treated with varying concentrations of each compound, either individually or in combination. Fixed molar ratios of baicalein/metformin were 1:250 for HEC-1A cells and 1:2000 for RL95-2 cells. Cell viability was assessed after 72 h. The combination treatment resulted in a synergistic reduction in cell viability in both cell lines, as demonstrated in Figure 6, indicating enhanced anti-tumor activity when baicalein and metformin were used together.

To elucidate the molecular mechanisms driving the observed synergy, we examined the effects of baicalein and metformin, individually and combined, on the key signaling pathways in HEC-1A endometrial cancer cells. As shown in Figure 6C, the combination treatment significantly increased the expression of DDIT4, enhanced AMPK activation, and reduced phosphorylation of S6 at 24 h, compared to either agent alone, suggesting that the combined treatment more effectively modulates the AMPK/PI3K/mTOR pathway.

## 3. Discussion

Given the advanced age and frequent comorbidities, such as obesity, diabetes, and hypertension, among patients with endometrial cancer, the development of non-toxic therapeutic strategies is of critical importance [2,3]. In this study, we investigated the anti-tumor effects of baicalein in human endometrial cancer, a disease often associated with dysregulation of the PI3K/AKT/mTOR signaling pathway, particularly due to frequent loss of PTEN function.

Our findings show that baicalein significantly reduces endometrial cancer cell viability in a dose-dependent manner, likely through the induction of apoptosis. Preliminary results) showed that baicalein increased the cleavage of caspase-3 and poly (ADP-ribose) polymerase (PARP), both markers of apoptotic activity. In addition, treatment with baicalein increased the expression of DDIT4 (a negative regulator of mTOR), enhanced AMPK phosphorylation, and reduced phosphorylation of downstream effectors S6K1 and S6, consistent with prior studies in breast and ovarian cancer. Despite these findings, the precise molecular mechanisms underlying baicalein’s effects remain defined. Future studies employing advanced target identification and validation approaches, such as quantitative proteomics, phosphoproteomic profiling, and PROTAC-based chemical probes [42], will be essential for elucidating its direct molecular targets and clarifying its mode of action.

Metformin, a drug currently under clinical investigation for the treatment of endometrial cancer [31,32], is shown here to enhance the anti-tumor efficacy of baicalein. As a well-established AMPK activator and mTOR inhibitor, metformin is widely prescribed for type 2 diabetes, with over 200 million users worldwide [40]. Epidemiological data suggest a protective effect against cancer in diabetic populations, prompting interest in its repurposing for oncology [40]. Consequently, considerable efforts have been directed toward evaluating its efficacy across various cancers, including endometrial cancer, through large randomized clinical trials [29,43]. However, these studies have yielded inconsistent and inconclusive results, highlighting the need for further clinical investigation to fully elucidate metformin’s role in cancer therapy, both as a monotherapy and in combination regimens.

Preclinical studies have demonstrated metformin’s efficacy in multiple cancer types, including lung, breast, colon, melanoma, and endometrial cancers, primarily through activation of the AMPK signaling pathway [24,25]. In our study, the IC_50_ of metformin in endometrial cancer cells was relatively high (>1 mM), consistent with previous in vitro findings in other cancers. Given that circulating plasma concentrations of metformin in vivo typically remain below 100 µM [43], this suggests that metformin alone may have limited direct anti-tumor efficacy at clinically achievable doses. However, when combined with baicalein, metformin significantly reduced cancer cell viability at a much lower IC_50_ and more effectively inhibited the AMPK/PI3K/mTOR pathway, indicating that baicalein sensitizes cancer cells to metformin’s effects. This combination may overcome the limitations of metformin as a single agent by allowing for effective tumor suppression at more physiologically relevant doses.

Although our study demonstrated that the combination of baicalein and metformin exerts stronger inhibitory effects on the AMPK/PI3K/mTOR pathway and a synergistic suppression of cell growth, the specific molecular mechanisms underlying this synergy remain to be elucidated. Key questions, such as whether the two compounds activate AMPK through distinct mechanisms, whether cross-regulation occurs among upstream and downstream signaling components, or whether there are synergistic effects on other related pathways (e.g., the JAK/STAT3 pathway), remain unresolved.

The ability of baicalein to enhance metformin’s therapeutic effects has also been demonstrated in metabolic disease models. Research has shown that combining metformin with *Scutellaria baicalensis* (SB) can significantly improve outcomes in individuals with type 2 diabetes while mitigating side effects associated with metformin, such as diarrhea, nausea, and vomiting by enabling lower effective doses of metformin. This combination has been shown to enhance glucose tolerance, reduce inflammation, and positively influence gut microbiota composition [44]. Baicalein, a key compound in SB, has also been shown to enhance metformin’s efficacy by improving glucose regulation, reducing inflammation, promoting healthier lipid metabolism, and supporting gut microbiota balance in prediabetic mouse models [44,45,46]. Given that baicalein is well tolerated at doses as high as 2.8 g/day, the doses used in combination therapy (40–160 mg/kg/day in mice, roughly equivalent to 0.2–0.78 g/day for a 60-kg human) are considered safe and practical for oral administration as dietary supplements [32,47,48].

In conclusion, our results demonstrate that baicalein, a natural flavonoid, reduces cell viability in endometrial cancer cells by inhibiting the AMPK/PI3K/mTOR pathway and enhances the anti-proliferative effects of metformin. Given its natural origin and favorable safety profile, baicalein represents a promising candidate for further preclinical development and clinical evaluation, particularly in combination with metformin. Future in vivo studies and clinical trials will be essential to validate these results, optimize dose, and identify patient populations most likely to benefit from this combinatorial therapeutic approach.

## 4. Materials and Methods

### 4.1. Reagents

Baicalein was synthesized at the Chemical GMP Synthesis Facility at the Beckman Research Institute of the City of Hope National Medical Center (Duarte, CA, USA) (Figure S1). Metformin was purchased from Sigma-Aldrich (St. Louis, MO, USA). The antibody against DDIT4 (1:3000 dilution) was obtained from ProteinTech Group (Rosemont, IL, USA), and the antibody against IRF1 (H205) (1:1000 dilution) was from Santa Cruz Biotechnology (Dallas, TX, USA). Antibodies against phospho-AKT (Ser473) (1:1000 dilution), total AKT (1:1000 dilution), phospho-S6K1 (Thr389) (1:1000 dilution), total S6K1 (1:1000 dilution), phospho-S6 (Ser235/236) (1:6000 dilution), total S6 (1:6000 dilution), phospho-STAT3 (Tyr705) (1:1000 dilution), total STAT3 (1:1000 dilution), phospho-AMPK (Thr172) (1:1000 dilution), total AMPK (1:1000 dilution), β-actin (1:1000 dilution), and GAPDH (1:8000 dilution) were obtained from Cell Signaling Technology (Danvers, MA, USA).

### 4.2. Cell Culture

Human endometrial cancer cell lines HEC-1A and RL95-2 were obtained from the American Type Culture Collection (ATCC, Manassas, VA, USA), while ECC-1 cells were kindly provided by Dr. Carlotta Glackin from City of Hope National Medical Center (Duarte, CA, USA). HEC-1A cells were cultured in McCoy’s 5A Medium, RL95-2 cells in DMEM/F12 Medium, and ECC-1 cells in RPMI-1640 Medium. All media were supplemented with 10% fetal bovine serum (FBS) and 1% penicillin/streptomycin. Cells were maintained at 37 °C in a humidified atmosphere containing 5% CO_2_ [11].

### 4.3. Cell Viability Assays

Cells were seeded in 96-well plates with 100 μL of growth medium per well and incubated for 24 h. Subsequently, cells were treated with DMSO (vehicle control, less than 0.1%) or the indicated drugs at various concentrations and incubated for an additional 2–3 days. Cell viability was measured using the MTS assay (Promega, Madison, WI, USA) following the manufacturer’s instructions [11]. Results are representative of 3 or more preparations. Data are expressed as the ratio to control treated with vehicle (DMSO). The half-maximal inhibitory concentration (IC_50_) was calculated using Calcusyn software V2 (Biosoft, Ferguson, MO, USA) [49].

### 4.4. Determination of Combination Index

The combination index (CI) for synergy was calculated using the Chou-Talalay method with Calcusyn software [49]. CI values were determined at the fraction affected (a fraction of cell viability inhibited) as well as at effective concentrations EC50, EC75, and EC90. A CI value less than 1 indicates synergy, a value greater than 1 indicates antagonism, and a value equal to 1 denotes an additive effect.

### 4.5. Immunoblot Analysis

Cells were cultured overnight in complete medium and subsequently treated with either dimethyl sulfoxide (DMSO, less than 0.1%) or baicalein at the indicated concentrations for the specified durations. Following treatment, cells were lysed using RIPA buffer (Thermo Scientific, Waltham, MA, USA) containing Halt protease and phosphatase inhibitors (Thermo Scientific). Proteins were quantified using BCA protein assay reagent (Thermo Scientific). Equal amounts of protein were then separated by SDS-polyacrylamide gel electrophoresis, and Western blotting was performed as previously described [11].

### 4.6. Animal Models

All animal studies were carried out under protocols approved by the Institutional Animal Care and Use Committee at City of Hope (IACUC#11013; approval date: 10 May 2023) and complied with ARRIVE (Animal Research: Reporting of In Vivo Experiments) guidelines. The mice were housed under standard laboratory conditions, including 12-h light/dark cycle, a temperature of 22 ± 2 °C, and relative humidity 51 ± 5% with ad libitum access to food and water. HEC-1A cells (2 × 10^6^) were inoculated in the flank of 6- to 8-week-old female athymic nude mice (National Cancer Institute). After the tumors were palpable, mice were randomized by an independent investigator into two groups (*n* = 4 per group) to receive either vehicle or baicalein (80 mg/kg). Treatments were given daily by intraperitoneal (i.p.) injection. Tumor volumes were assessed using calipers twice a week and determined using the formula (Width)^2^ × Length × 0.5^2^. Mice were also monitored twice a week for body weight and any adverse effects. To minimize potential confounding factors, the order of treatments and measurements was kept consistent throughout the study. Humane endpoints were predefined as a tumor diameter exceeding 20 mm, the presence of tumor ulceration, or a body weight loss greater than 20%. Animals meeting any of these criteria were humanely euthanized and excluded from further analysis. Two mice in the vehicle control group developed tumor ulceration and were excluded from analysis on days 32 and 36, respectively.

### 4.7. Statistical Analysis

All experiments were performed in triplicate or more. Data are presented as the mean ± standard deviation (SD). Statistical comparisons were made using Student’s t-test, and a *p* value of <0.05 was considered statistically significant. Data analysis was conducted using GraphPad Prism 10 (GraphPad Software, San Diego, CA, USA).

## 5. Conclusions

Baicalein, a natural flavonoid, shows potent anti-tumor activity against endometrial cancer by inhibiting cell proliferation and suppressing the AMPK/PI3K/mTOR pathway. Combined treatment with metformin produces synergistic effects, highlighting baicalein as a promising, low-toxicity therapeutic candidate for endometrial cancer.

## Data Availability

The original contributions presented in this study are included in the article. Further inquiries can be directed to the corresponding author.

## Supplementary Materials

The following supporting information can be downloaded at: https://www.mdpi.com/article/10.3390/ijms262211061/s1. Reference [50] is cited in the supplementary materials.

## Abbreviations

The following abbreviations are used in this manuscript:

PI3K	Phosphoinositide 3-kinase
AKT	Protein Kinase B
mTOR	Mammalian target of rapamycin
S6K	S6 kinase
DDIT4	DNA-damage-inducible transcript 4
IRF-1	Interferon regulatory factor
PTEN	Phosphatase and tensin homolog
AMPK	AMP-activated protein kinase
STAT3	Signal transducer and activator of transcription 3
SB	*Scutellaria baicalensis*
CI	Combination Index
IC_50_	Half-maximal inhibitory concentration
