# Supplementary material for: Therapeutic Potential of Baicalein in Endometrial Cancer: Suppression of mTOR Signaling and Synergy with Metformin
Source: Int J Mol Sci. 2025 Nov 15;26(22):11061. doi: 10.3390/ijms262211061 (PMC12652256; doi:10.3390/ijms262211061)
Supplement: Supplementary file 1 [file ijms-26-11061-s001.zip › ijms-3936390-supplementary.pdf]

Baicalein (5,6,7-Trihydroxyflavone)

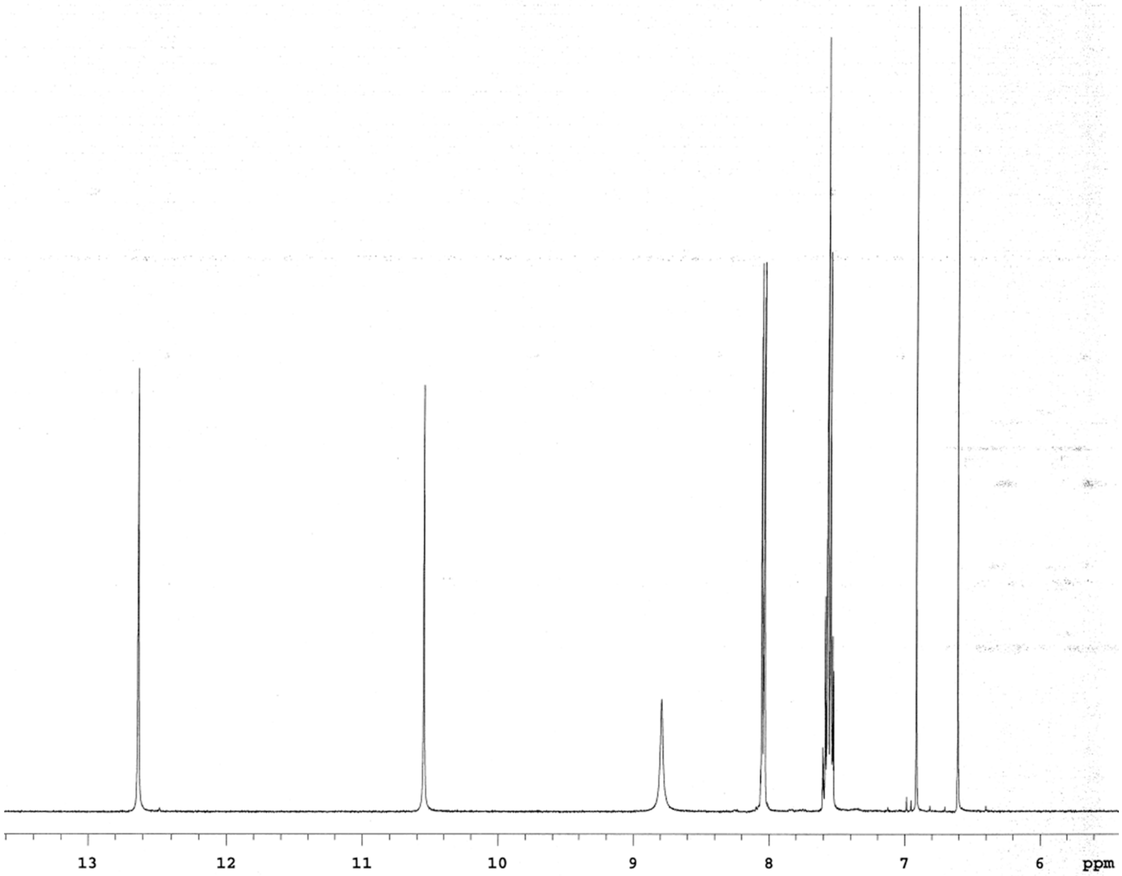

Figure S1. ^{1}H Spectra for Baicalein

^{1}H-NMR (400 MHz, DMSO-d6):

δH 12.64 (s, 1H, 5-OH), 10.56 (s, 1H, 7-OH), 8.80 (s, 1H, 6-OH), 8.06–8.04 (m, 2H, H-2',6'), 7.59-7.53 (m, 3H, H-3',4',5'), 6.92 (s, 1H, H-3), 6.61 (s, 1H, H-8). ^{1}H-NMR data obtained at City of Hope is consistent with the ^{1}H-NMR data for Baicalein found in the literature (*Wang et al.*, 2015)

Reference: Wang, S.-H., Chen, C.-H., Lo, C.-Y., Feng, J.-Z., Lin, H.-J., Chang, P.-Y., Yang, L.-L., Chen, L.-G., Liu, Y.-W., Kuo, C.-D., & Wu, J.-Y. (2015). *Synthesis and biological evaluation of novel 7-O-lipophilic substituted baicalein derivatives as potential anticancer agents. Med. Chem. Commun.* **2015**, *6*, 1864–1873. https://doi.org/10.1039/C5MD00163C

HPLC Data:

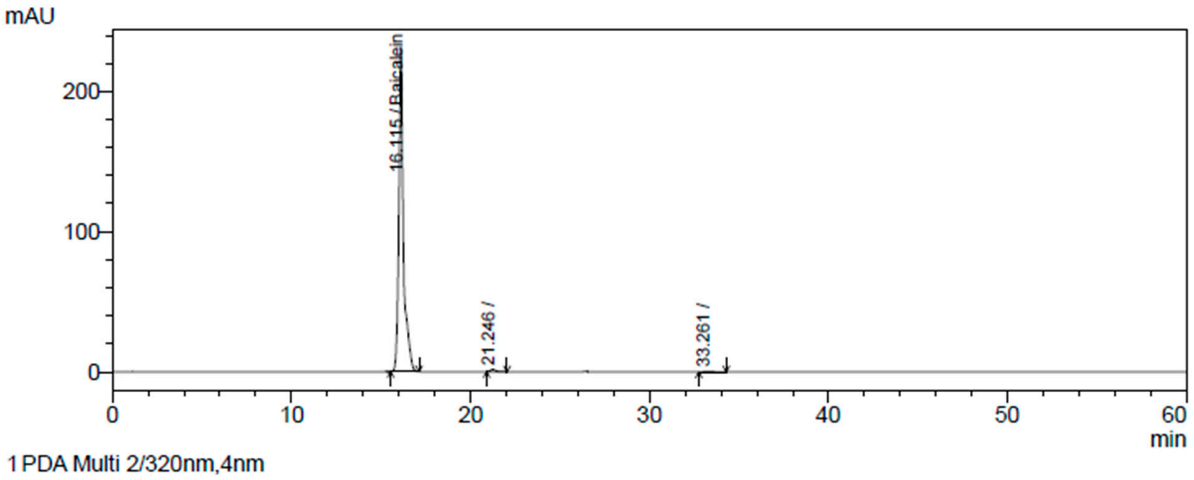

<Zoomed Chromatogram>

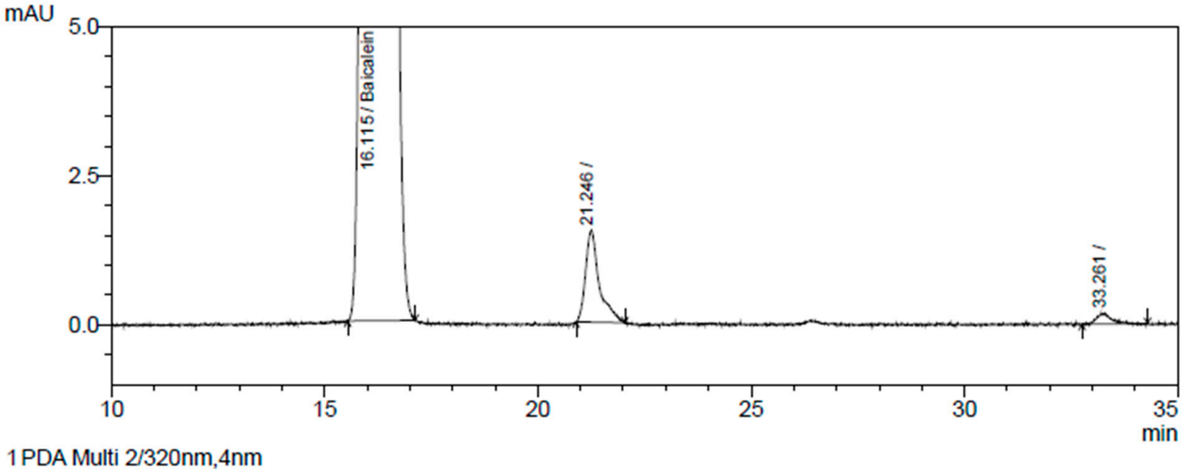

<Peak Table>

PDA Ch2 320nm

Peak#	Name	Ret. Time	Area	Area %
1	Baicalein	16.115	4138459	99.08
2		21.246	34423	0.82
3		33.261	4212	0.10
Total			4177094	100.00

HPLC method conditions: Detector: PDA, Wavelength: 320 nm, Flow rate: 2.0 mL/min, Column: Waters Atlantis T3 150 x 4.6 mm (part no. 186003747), Mobile phase A (MPA) – 0.1% TFA in water, & Mobile phase B (MPB) - 0.1% TFA in acetonitrile. Binary mobile phase system: MPA (80%-42%) & MPB (20%-58) over 55 mins.
